# Oxidative stress and leaf senescence

**DOI:** 10.1186/1756-0500-4-477

**Published:** 2011-11-02

**Authors:** Hatami Gigloo Sedigheh, Mahdi Mortazavian, Dariush Norouzian, Mohammad Atyabi, Azim Akbarzadeh, Keyvan Hasanpoor, Masoud Ghorbani

**Affiliations:** 1 Pasteur Institute of Iran (Research & Production complex), Department of Research and Development, 25th km Tehran Karaj Highway, Tehran, Iran; 2 University of Tehran, College of Aburaihan, Department of Agronomy and Plant Breeding, Pakdasht, Iran; 3 Pasteur Institute of Iran, Department of Biotechnology, Tehran, Iran; 4 University of Azad, Department of Horticulture, Takestan, Iran

**Keywords:** Senescence, Rubisco, reactive oxygen species, paraquat, photosynthesis, fluorescence

## Abstract

**Background:**

Senescence is an important developmental process that leads to the cell death through highly regulated genetically controlled processes in plants. Biotic and abiotic Oxidative stresses can also artificially induce senescence and increase the production of reactive oxygen species (ROS) specifically in chloroplast. One of the important oxidative stresses is paraquat that induces deviation of electron from photosynthesis electron chain and lead to the production of more ROS in chloroplast. Plants have evolved special adoptive mechanism to reallocate nutrient to reproductive and juvenile organs in senescence and different oxidative stresses. Rubisco seems to be the most abundant protein in plants and is involved in many changes during senescence.

**Results:**

In the present study, the effects of ROS on Rubisco during senescence and oxidative stresses were evaluated by measuring photosynthesis factors such as net photosynthesis rate (Pn), stomatal conductance (G), evaporation rate (E), intra cellular CO_2 _concentration (Ci), fluorescence and total protein during three stages of development. Our results showed that in paraquat treated plants, CO_2 _assimilation is the most effective factor that refers to Rubisco damages. The highest correlation and regression coefficient belonged to Ci, while correlation coefficient between photosynthesis rate and total protein was much smaller.

**Conclusion:**

It appears in the early stage of oxidative stresses such as exposing to paraquat, ROS has the most effect on Rubisco activity that induces more susceptibility to Rubisco specific protease. Moreover, Rubisco deactivation acts as an initiative signal for Rubisco degradation.

## Background

Senescence is a genetically programmed sequence of biochemical and physiological changes that occurs during the plant life. Plants have evolved mechanism by which the leaf senescence is induced by many environmental stresses to reallocate nutrients to reproductive organs [1].

One of the environmental stresses that can artificially induce senescence in plant is exposure to paraquat, a nonselective herbicide from bipyridylinum family that is used broadly to control the growth of broad-leaved weeds and grasses. Because of its relatively low cost, ease of use and effectiveness, paraquat has become one of most important means of weeds control over the last 50 years although it might have undesired effects on non-targeted crops that are meant to protect.

Paraquat has many direct and indirect effects on living organisms. When Paraquat is sprayed on leaves of green plants, it moves through cuticle into the cells and into the chloroplasts where photosynthesis is happening and interferes in photosynthesis process. Similar to other members of bipyridylium family, Paraquat inhibits photosystem I, by intercepting electrons (electron diversion) from one of the iron-sulfur protein electron acceptors in the normal electron transport sequence [2]. By blocking and accepting electrons from early acceptors of photosystem I, the divalent paraquat/diquat cation (+2charge) changes to an unstable "free radicals" (the cation is reduced). These free radicals are not toxic to plant, but since the molecule tends to become stable (the normal cationic state), the free radicals are re-oxidized (auto oxidation) in the presence of oxygen and water to yield the original ion. During this oxidation process, electrons (e^-^) are transferred to molecular oxygen (oxygen is reduced) and superoxide anion radicals (O2^-^) are produced. Superoxide radicals are then enzymatically altered to other oxygen species [3]. Therefore during senescence and any environmental stresses that may ultimately lead to senescence, large quantity of reactive oxygen species produces [4].

In fact, senescence is a defense or adoptive mechanism that allows plant to complete its life cycle even under stressful condition [1]. Therefore, plants remobilize nitrogen from old leaves to reproductive and young organs [1,5]. The most abundant protein in plants that contain approximately 40% of total leave proteins [5] and 20-30% of total leaf nitrogen [6] is ribulose 1, 5- bisphosphate carboxylase oxygenize (Rubisco). It seems that this enzyme is nitrogen budget of plants.

Rubisco that is a critical enzyme of Calvin cycle in stroma of chloroplast and catalyzes two competing reaction, photosynthetic CO_2 _fixation and photorespiratory carbon oxidation respectively, is one of the early proteins that are broken down during senescence and oxidative stresses [1]. To date many proteases have been known to have proteolytic activity on Rubisco during senescence and oxidative stresses. Roberts et al. [7] was the first to suggest that oxidative modification of Rubisco may target this protein for subsequence degradation. Serine protease in chloroplast has an important role in Rubisco degradation in senescence. Chloroplasts are major site of protein degradation during senescence [8].

It has been shown that under abiotic stress conditions, the increased cystein endoproteinase activity is potentially involved in response to stress [1,9]. Nakano et al. [10] has found that reactive oxygen species may triggers the site specific degradation of the large subunit of Rubisco (LSU) in lysates of chloroplast in high light. The cross-linked LSU has been found to be more sensitive to proteases [11]. Pell et al. [12] noted that ozone may induce oxidative modification of Rubisco leading to subsequent proteolysis. Eckardat and Pell [13] also suggested that oxygen free radicals in leaves exposed to O_3 _could bring about leaf ageing in *Solanum tuberosum L *and enhance degradation of Rubisco in mature leaves.

Reactive oxygen's have many direct and indirect effects on Rubisco and herbicide. It has been shown that reactive oxygen radicals can directly induce Rubisco fragmentation to 37 and 15 KD polypeptides [14,15]. As well, reactive oxygen species may triggers the site specific degradation of the large subunit (LSU) of Rubisco in lysates of chloroplast in the presents of light [5]. Desimone et al. [14] also investigated the effects of oxidative stresses in isolated chloroplast of barley and suggested that oxidative stresses can induce partial degradation of Rubisco. Luo et al. [16] also found that Rubisco large subunit is cleaved by reactive oxygen species at the metal binding site, close to the active site of the molecule.

One of the mechanisms involved in stimulating cyclic electron flow is phosphorylation of light-harvesting chlorophyll complex of PSII (LHCII). Phosphorylated LHCII moves from the adjacent thylakoid regions, where PSII is located, to the further regions, where PSI is located [2]. The phosphorylated LHCII becomes energetically disconnected from PSII core complex (slowing its turnover rate) and energetically coupled to a PSI core (increasing its turnover rate). This process requires a specific polypeptide within PSI [17]. This is a well-known state transition [18] that is known to accompany an increase in cyclic electron flow around PSI and response to heat [2].

Variarion in ascorbate peroxidase activity is another main factor that is affected by paraquat and oxidative stress. Reports have shown that paraquat decreases the activity of ascorbate peroxidase, resulting in the accumulation of hydrogen peroxide in the chloroplast [19,20].

Zhou et al [21] in 2006 indicated that H_2_O_2 _accumulation in chloroplast was negatively correlated with the initial Rubisco activity and photosynthetic rate. Feller et al. [5] demonstrated the importance of reactive oxygen in cleaving the large subunit or modifying the Rubisco to become more susceptible to proteolysis.

Reactive oxygen may also indirectly affect the other macromolecules resembling DNA and proteins and may induce more activation of Rubisco specific proteases in the translational or post translational stage of expression that lead to more Rubisco degradation. Kato et al. [22] showed that a reduction of DNA copy number during senescence could initiate the activation of CND41 in chloroplast. Casano et al. [23] also demonstrated that the activity of thylakoid-bound endopeptidase (EP) increased under both photo oxidative environmental conditions and treatment with an OH-generating system in oxygen-induced proteolysis in chloroplasts of oat.

The objective of this project was to quantifying the special effects of oxidative burst on the activity and content of Rubisco as a result of senescence and oxidative paraquat stress. Therefore, the leaf gas exchange and chlorophyll fluorescence parameters for determination of photosynthetic and Rubisco activity in different stage of development followed by paraquat treatment in wheat plants were measured. We also examined the changes of total protein and Rubisco contents.

## Methods

### Plant material and growth conditions

Seeds of Wheat (*Triticum aestivum *L., "Roshan") were germinated in pots of vermiculate in greenhouse with a photo period (day light) between 8 and 24 h and a dark period (night) between 24 and 8 h, at a day/night temperature of 22/17°c respectively. A factorial design (3 × 3) was used in which the developmental stages (pre-anthesis, post-anthesis, post-maturation) were considered as one factor and paraquat (methyl viologen, 1, 1'-dimethyl-4, 4'-bipyridinium dichloride; Sigma, St Louis, MO, USA) doses (0, 300 μM, 600 μM) were considered as another factor.

Plant were sprayed thoroughly with different doses of paraquat diluted in 4% Tween 80 in distilled water and exposed to light for 3 hours. The control plant was only sprayed with 4% Tween in distilled water and exposed to light for 3 hours as well. Immediately after the light exposure period, the gas exchange and fluorescence parameters were measured and leaves were harvested and store in liquid nitrogen.

### DNA extraction

Plant material including leaves of wheat were sprayed perfectly with paraquat doses (0, 300 μM, 600 μM) in distilled water containing 4% Tween 80 and exposed to light for 3 h. For determination of DNA damages along with senescence in plants, juvenile (perfectly green) and senescence (yellow) leaves of every plant were used. Total DNA of plant samples were extracted according to the Cetyl Trimethyl Ammonium Bromide (CTAB) *_*activated charcoal protocol of Krizman et al. [24] with some modifications. Briefly, for inhibition of DNA degradation during extraction, fresh leaves samples (0.5 g) were cut with blade to small fragments (1 × 1 mm). After addition of 0.6 g CTAB, .03 g PVP and .015 g activated charcoal, leaves were softly homogenized in 3 ml extraction buffer containing 100 mM Tris-HCl (pH 8), 2.0 M NaCl, 20 mM EDTA (pH 8). The homogenate was then transferred into a microcentrifuge tube and Incubated at 55°C for 30 min with frequent agitation, avoiding the suspension to settle. It was then cooled down to room temperature and Centrifuged at 16000 g for 10 min at room temperature. The supernatant was then transferred to a new tube, followed by addition of 1 volume of chloroform-isoamylalcohol (4% (v/v) to the supernatant and vortexed thoroughly. Samples were then centrifuged at 16000 g for 10 min at room temperature and the aqueous (upper) phase was transferred to a new tube. The supernatant was then transferred to a new tube and 0.45 volume of isopropanol was added and mixed by inversion. The mixture was incubate at 25°C for 1 hour and centrifuged at 700 g for 10 min at room temperature. The supernatant was discarded and the remaining pellet was washed by adding 1 mL of wash buffer followed by vortexing. After centrifugation at 900 g for 10 min at room temperature and discarding the supernatant the pellet was air dried and resuspended in 50 μL of distilled water.

The extracted DNA was then used as a template for amplification of Rubisco gene in a polymerase chain reaction (PCR) using the forward primer 5'- TGG ATT CAA AGC TGG TGT TA- 3' and the reverse primer 5'- TAC TCG ATT AGC TAC GGC AC- 3' according to the NCBI accession number AM087200. The PCR reaction was performed with 35 cycles and annealing temperature of 60°C. The extension and denaturing temperatures were set at 72°C for 1 min and 94°C for 30 second as quoted in most standard protocols.

### Gas exchange and chlorophyll fluorescence measurements

Leaf gas exchange and chlorophyll measurement were performed using a portable photosynthetic system (IRGA, ADC. Ltd UK) and a chlorophyll fluorometer (OS30, ADS. Ltd UK) at 10:30 AM before paraquat treatment and four hours after paraquat treatment at 2:30 PM, respectively. To avoid the occurrence of photosystem II (PSII) during the measurement of fluorescence in the leaf sample, a small piece of leaf was wrapped with aluminum foil approximately 30 minutes before placing in the measuring chamber to detect the photosynthesis factors including photosynthesis rate (Pn), stomatal conductance (G), evaporation rate (E), intracellular CO_2 _concentration (Ci), fluorescence and total protein and Rubisco content.

### Total protein extraction and assay

All operation were performed at 4°C. Leaves (1 g) were homogenized with a mortar in 3 ml ice cold 50 mM potassium phosphate (pH 7) containing Sodium meta bisulfate 1 mM and 100 mg g^-1 ^of Polyvinyl pyrrolidone (PVP). The homogenate was then collected and mixed with 1/3 glycerol 50% and after freezing in liquid nitrogen and stored at -80°C. Protein content was measured according to the Bradford [25] method using bovine serum albumin as standard. The soluble protein extract, specifically Rubisco large subunit (LSU) of approximately 55 kilo Dalton, of leaves was quantitated using the method of Laemmli [26] and analyzed by SDS-PAGE on 11.5% acrylamide gel followed by staining with coomassie brilliant blue (CBB).

### Ascorbate Peroxidase Activity determination

Ascorbate peroxidase activity (APX) was determined using Nakano and Asada [27] method. Briefly, enzyme assay APX activity was determined in a lmL reaction mixture containing 50 mM potassium phosphate (pH 7), 0.5 mM ascorbate, and 0.1 mM hydrogen peroxide. The reaction was initiated by addition of hydrogen peroxide, and oxidation of ascorbate was followed by the decrease in A at 290 nm. One unit of APX activity is defined as the amount of enzyme that oxidizes 1 gmol of ascorbate per min at room temperature under the above conditions. Oxidation of alternate electron donors was measured in the same assay mixture as that used for ascorbate, but ascorbate was replaced by 10 mM guaiacol, the reaction was initiated by addition of 0.1 mM or 0.5 mM hydrogen peroxide, and substrate oxidation was followed by the decrease in the A430 and A470, respectively. HRP (EC 1.11.1.7, Sigma) activity was determined in the same reaction mixture used for APX activity, but 2 mM hydrogen peroxide was included. One unit of HRP is defined as the amount of enzyme that oxidizes 1 gmol of guaiacol per min under the above conditions.

### Statistical analysis

Results are presented as mean ± SD. Statistical analysis used MSTATC software for analysis of variance followed by Student-Newman-Keuls post hoc test. Significant differences were assessed at *P *< 0.05.

## Results

### Photosynthesis rate (Pn)

**Figure 1:**
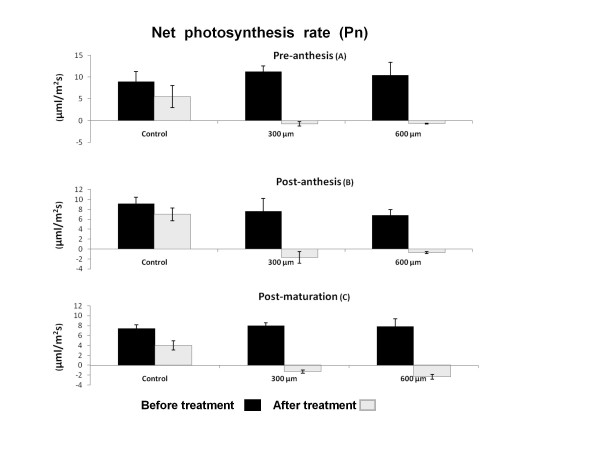
**Effect of paraquat on Net Photosynthesis Rate (Pn) in wheat leaves**. Pn was quantitated before and after treatment with three different concentration of 0, 300 and 600 μM of paraquat. Paraquat treatment significantly decreased Net Photosynthesis Rate in all stages A, B, and C of development (*P < 0.01*).

During normal stage of leaf growth in wheat plant, there is no significant differences in photosynthesis rate between pre-anthesis, post-anthesis and post maturation stages although it appears that by increasing the age, Pn rate decreases significantly (*P *< 0.05) (Figure 1A). This reduction is mainly observed among three developmental stages in control (time zero) after treatment. Paraquat treatment hindered the photosynthesis rate in a dose of 300 μM. As shown in Figure 1B, 3 hours after paraquat treatment, photosynthesis rate decreased significantly. The lowest Pn is observed in the presence of 300 μM of paraquat in the post anthesis plant, although, there was no significant difference among different growth plant stages in the presence of 300 μM of paraquat (Figure 1C). By increasing the paraquat dose to 600 μM, a further decrease of photosynthesis rate was observed *(P *< 0.01). This dose caused the highest decrease in Pn at post-maturation stage (Figure 1C).

### Intracellular CO_2 _concentration (Ci)

**Figure 2:**
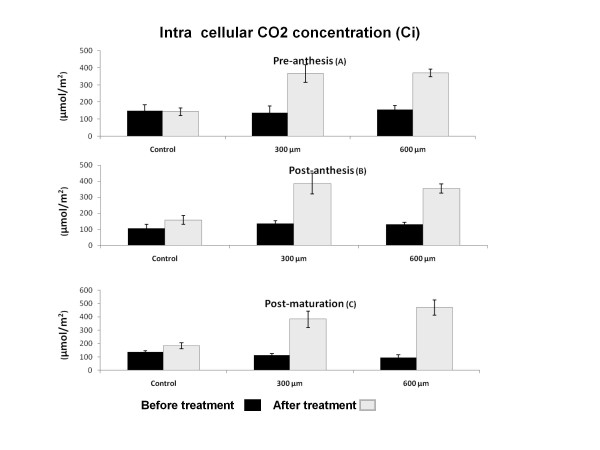
**Effect of paraquat on Intra Cellular CO2 concentration (Ci) in wheat leaves**. Ci was quantitated before and after treatment with three different concentration of 0, 300 and 600 μM of paraquat. Paraquat treatment significantly increased Ci in all stages A, B, and C of development (*P < 0.01*).

There was no significant difference in the intracellular concentration of CO2 in the control group at time zero. However, 3 hours after treatment with either 300 μM or 600 μM of paraquat Ci increased significantly in all stages of development including pre-anthesis, post-anthesis and post-maturation stages (*P *< 0.01) (Figure 2A-C). It is notable that there is a significant difference in Ci during development from pre-anthesis to post-anthesis and post-maturation stage in untreated plants as well (*P *< 0.05) (Figure 2A-C)

### Stomatal conductance (G)

**Figure 3:**
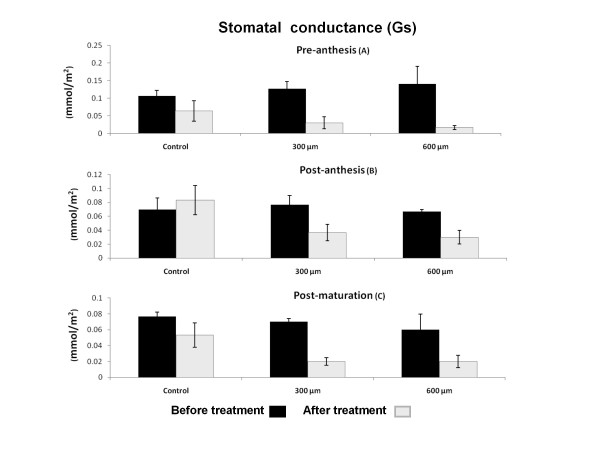
**Effect of paraquat on Stomatal Conductance (G) in wheat leaves**. G was quantitated before and after treatment with three different concentration of 0, 300 and 600 μM of paraquat. Paraquat treatment significantly decreased G in all stages A, B and C of development (*P < 0.01*).

In wheat plant like most other plants, G is normally decreased when the plant gets older [28]. Our results also showed a significant decrease in the rate of G during the developmental stage of the plant (Figure 3A). Paraquat treatment, on the other hand, brought about an additional 1% decrease in G rate in all three stages of development (Figure 3B, C and 3D). There was no significant difference between the effect of 300 μM and 600 μM paraquat on G rate in plants.

### Evaporation rate (E)

**Figure 4:**
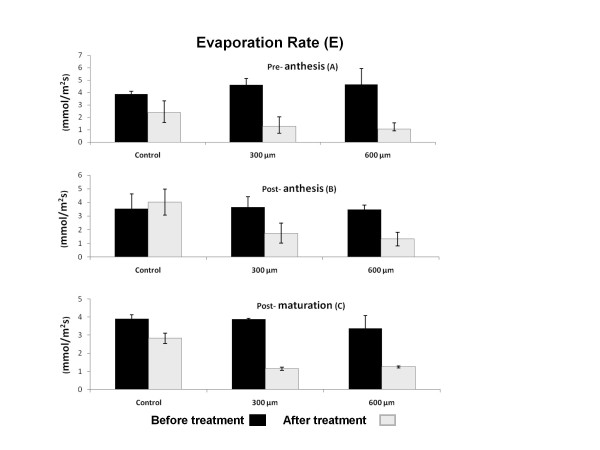
**Effect of paraquat on Evaporation Rate (E) in wheat leaves**. E. was quantitated before and after treatment with three different concentration of 0, 300 and 600 μM of paraquat. Paraquat treatment significantly decreased E in all stages A, B and C of development (*P < 0.01*).

Measurement of evaporation rate showed the same pattern as G and decreased significantly throughout the development of plant (data not shown). Paraquat treatment induced a significant decrease in E rate in all three stages of development (*P *< 0.01) (Figure 4A, B, and 4C). There was a significant decrease of 0.05 between paraquat doses of 600 μM and 300 μM as well.

### Fluorescence parameters

**Figure 5:**
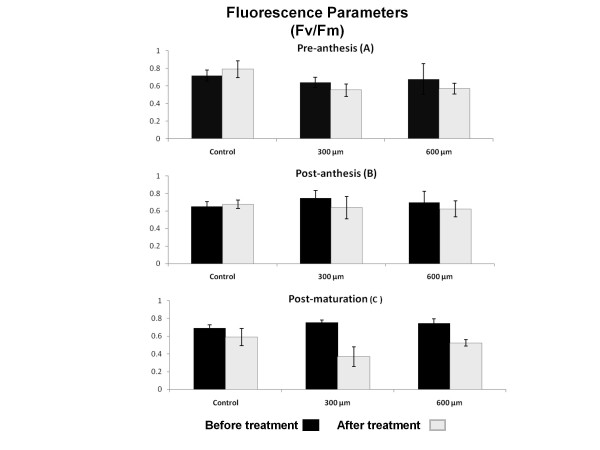
**Effect of paraquat on Fluorescence Parameters (Fv/Fm) in wheat leaves**. Fv/Fm was quantitated before and after treatment with three different concentration of 0, 300 and 600 μM of paraquat. Paraquat treatment significantly decreased Fv/Fm only in stage C of development (*P < 0.01*) and had no significant effect in stage A and B.

Measurement of various photosynthesis fluorescence parameters such as F0 (fluorescent in ground state), Fm (maximal value of fluorescence), T_1/2 _(time needed for F0 to rise to Fm) and Fv (Fm-F0)/Fm before and after treatment with paraquat was performed. There was no significant difference among any of these parameters during development of examined wheat plants. Our results show that, 3 hours after treatment with paraquat, only Fv/Fm parameters significantly decreased, whereas F0, Fm and T_1/2 _remained unchanged (*P *< 0.01). No difference was observed between the different concentration of paraquat (Figure 5A, B and 5C).

### Total soluble Protein and Rubisco content

**Figure 6:**
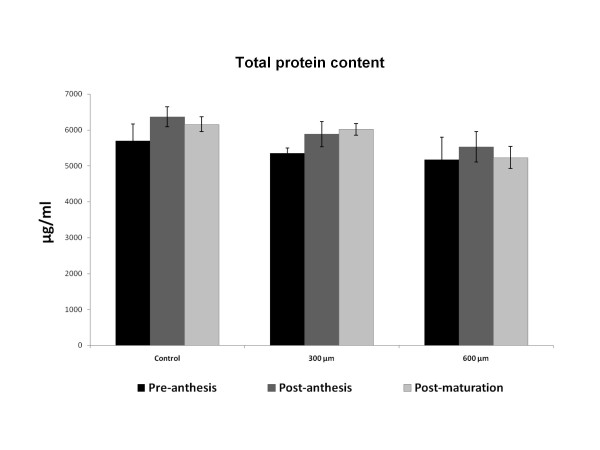
**Effect of paraquat on Total Protein Content in wheat leaves**. Treatment with paraquat induced no significant changes in wheat leaves.

**Figure 7:**
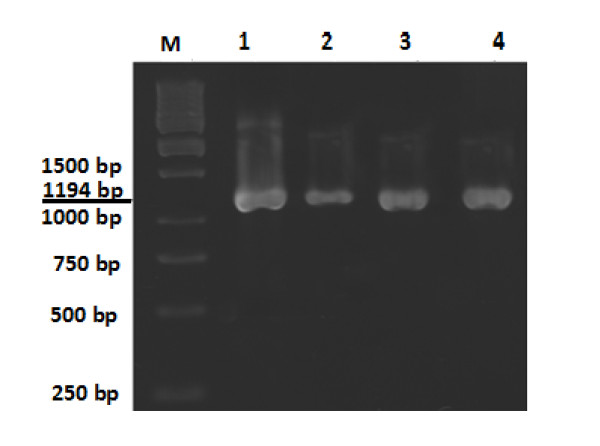
**Expression of Rubisco in wheat leaf at the gene level using specific forward (5'- TGG ATT CAA AGC TGG TGT TA- 3') and reverse (5'- TAC TCG ATT AGC TAC GGC AC- 3') primers for Rubisco**. 1) Pre-anthesis level, 2) Treated leaves with 600 μM paraquat, 3) Early stage of senescence in wheat leaves (green-yellowish leaves). 4) Advanced stage of senescence in wheat leaves (thoroughly yellow leaves). M) Molecular weight marker.

Quantification of total protein contents using Bradford assay [25] showed significant changes in protein concentration during different developmental stages after treating leaves with paraquat (Figure 6). Both paraquat doses of 600 μM and 300 μM had similar effects on reduction of protein contents in leaves samples. PCR analysis of chloroplast DNA also demonstrated similar results by reducing the DNA content after treatment with paraquat (Figure 7, lane 2). DNA content of chloroplast was also decreased in early and advanced stages of senescence in wheat leaves (Figure 7, lanes 3 and 4)

**Figure 8:**
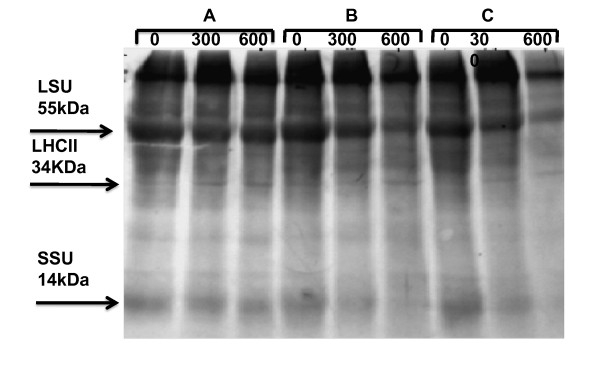
**SDS-PAGE analysis of Rubisco large subunit (LSU), small subunit (SSU) and light harvesting complex chlorophyll (LHCll) under influence of the oxidative stress**. A. Pre-anthesis, B. Post anthesis, C. Post-maturation. Detectable 55 kDa, 34 kDa, and 13 kDa protein bands correspond to Rubisco LSU, LHCll and SSU respectively in wheat samples. Wheat seeds were germinated on wet tissue paper in dark for 2 days and then transferred to a light/dark cycle (13 h light/10 h darkness). Wheat plants were grown until development stage (pre anthesis, post anthesis and post maturation), and were treated with paraquat at 0, 300 μM and 600 μM respectively. Each lane was loaded with 20 μl of wheat leaf extract containing an equal percentage (0.6%) of a leaf.

SDS-Page analysis of all three stages of development showed an easily detectable 37.6-66.2KD protein band corresponding to Rubisco. As shown in Figure 8, the expression of Rubisco in juvenile stage was higher as compared with post anthesis and post-maturation stages. Paraquat treatment also decreased the protein expression at 600 μM in both post anthesis and post-maturation stages. Paraquat seems to have no effect or little effect on pre anthesis stage of the development. Lower concentration of paraquat did not have any remarkable effect on different stages of development (Figure 8)

### 2- Ascorbate Peroxidase (APX) specific activity

**Figure 9:**
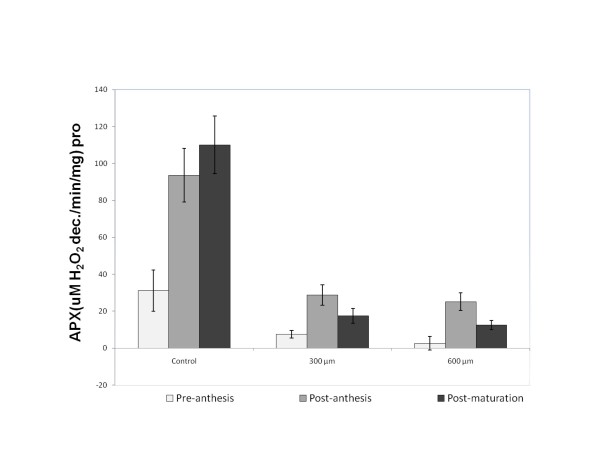
**Ascorbate peroxidase activity in wheat leaves treated with paraquat**.

The specific activity of ascorbate peroxidase was increased significantly during post anthesis and post maturation stages (*P *< 0.01), whereas, no significant changes of enzyme activity were observed in pre-anthesis stage. Both doses of paraquat (300 μM or 600 μM) had similar effects to induce a significant decrease of enzyme activity in treated leaves. (*P *< 0.01) (Figure 9).

## Discussion

A marked reduction in photosynthetic rate (Pn) in leaves during aging and paraquat treatments have been reported previously [29]. Donahue et al. [19] also reported that in pea plant, juvenile leaves have higher Pn [19]. Decreased Pn has also been reported to be dependent on stomatal or nonstomatl limitation [6,30]. Stomatal conductance (G) is one of the most important stomatal factors that controls the flooding of CO_2 _(the principle substrate of photosynthesis) to intracellular space. G measurement in this study has shown a remarkable reduction in aged and paraquat treated leaves. Great reduction of G (54%) in cucumber seedling treated with paraquat also reported by Xia et al. [31]. In 1979 of Wong et al. [3] discovered that G had a linear correlation to Pn under variety of environmental condition. In this study, we demonstrated a strong correlation between G and Pn (R^2 ^= 0.87) and their significant regression (*P <*0.05). It appears that G plays an important role in reduction of Pn during treatment with paraquat. Other investigators have shown that resistance to paraquat in conyza is because of failure in G and suppression of CO_2 _fixation due to stomatal closure caused by membrane damage and lose of water from the guardian cells [20].

E is another stomatal factor that can limit photosynthesis rate. Alteration in E factor is directly correlated with G ratio. Strong correlation between G and E (R^2 ^= 0.94) shows reduction in CO2 assimilation that could reduce the evaporation ratio during the development stages such as pre-anthesis and post-anthesis. Simultaneous reduction of G and E in other plants such as pea under oxidative stresses by paraquat has also been reported previously [32]. In fact, G can control the photosynthesis by acting directly on E or gas exchange through the photosynthetic tissue and CO_2 _availability in the assimilation sites of chloroplast [6]. Constant increment of Ci during developmental stages in this study could give us the idea that aged leaves are more sensitive to the changes during the day light. Although paraquat treated aged leaves are still sensitive to the day light, their sensitivity is significantly less than juvenile leaves treated with paraquat (Figure 2). Our findinG confirmed previous study by Xia et al. [31] that demonstrated the increase of Ci in paraquat treated Cucumis sativus. Strong correlation (R^2 ^= 0.92) and regression (85.3%) (Table 1) between Ci and Pn in one hand and decreasing of G that is limiting the Ci rate on the other hand suggest that the increase of Ci brinG about no consumption of CO2 by CO2 assimilation system. We suggest that CO2 concentration, supply of ATP and NADPH and the activity of Calvin cycle specially Rubisco are important factors in CO2 assimilation. ROS, on the other hand, can decrease CO2 assimilation capacity during oxidative condition with deactivation and fragmentation of Rubisco. Other studies have also shown that reactive oxygen species may trigger the site specific degradation of the large subunit of Rubisco in lysates of chloroplast in high light and low temperature 6 [10].

**Table 1:** correlation rate between total soluble protein, net photosynthesis rate (Pn), intra cellular concentration (Ci), stomatal condenses (gs), evaporation rate (E) and photosystem efficiency (F).

F	F	gs	Ci	Pn	Protein	
199/0	491/5	399/0	-334/0	389/0	1	Protein
507/0	827/0	877/0	-926/0	1	389/0	Pn
-445/0	-716/0	-750/0	1	-926/0	-334/0	Ci
536/0	948/0	1	-750/0	877/0	399/0	gs
513/0	1	948/0	-716/0	827/0	491/0	E
1	513/0	536/0	-445/0	507/0	109/0	F

Chlorophyll fluorescence parameters are none stomatal factors that effect on photosystem chain reaction [28]. Quantification of chlorophyll fluorescence parameters in this study indicated that there was no significant difference between F0 and T1/2 in aging and paraquat treatment. Since F0 was acquired from the fluorescence of antenna chlorophylls associated with PSI and PSII, paraquat treatments and aging had no significant effect on the chlorophyll fluorescence parameters. One of chlorophyll fluorescence parameters, Fm, reduced significantly after paraquat treatment in the third stage of development. This could be due to higher sensitivity of the leaves during post maturation stage to external environmental and chemical factors such as paraquat.

Analysis of maximum quantum efficiency of PSII (QF) before and after paraquat treatment between different stages demonstrated a significant decrease 3 hours after paraquat treatment that could be due to the damages of thylakoid sub-organelle membrane in sensitive leaves. It appears that these damages caused by stresses such as heat and light are age dependent whereas damages caused by paraquat are not. QF seems to act independently and does not alter photosynthetic rate although it effects directly on electron transfer chain, chlorophyll and thylakoid sub-organelles. It also appears that there is a significant correlation between QF and Pn (R^2 ^= 0.5). However, the regression between these two factors was insignificant. Although, treatment with paraquat induced significant damages to thylakoid as a chlorophyll fluorescence parameter, it had no effects on Pn rate.

Another important nonstomatal factor that strongly affects oxidative stresses and Pn changes is Rubisco. Quantification of total soluble protein and Rubisco content in aging and treated plants with paraquat revealed that the total protein increases in the stage 2 of plant development, as compared with other stages.

After that, the plant enters into senescence followed by reduction at total soluble protein specially Rubisco. Paraquat treatment significantly reduced total soluble protein in a dose dependent manner, as reported previously by Iturbe-Ormaetxe et al. [32] and Donahue et al. [19,32].

Comparison of Rubisco content on polyacrilamid gel electrophoresis also showed that the density of Rubisco (LSU) band in leaves treated with paraquat is less than control leaves. This result also indicated that the density of Rubisco band in aged leaves treated with paraquat is lesser which is due to the higher sensitivity of Rubisco (LSU) to paraquat treatments (Figure 8).

Correlation and regression analysis between total soluble protein and Pn demonstrated a significant correlation (R^2 ^= 0.38) but insignificant regression, suggesting that Pn cannot be an important factor to influence Pn alterations (Table 1). Since Rubisco is known as a non stomatal controlling factors for Pn, we expected it's concentration would affect Pn, however, our Results demonstrated that the decrease of Rubisco protein due to senescence and paraquat treatment had no significant effect on Pn reduction.

On the other hand we believe that Rubisco is more sensitive to ROS produced during the senescence and paraquat treated [10,14,15,33]. Our experiments also suggest that ROS will affect the catalytic site of Rubisco and induce its degradation before complete fragmentation which results in deactivation of Rubisco and decrease of Pn rate. Therefore, deactivation of Rubisco may be more important than any other photosynthesis factor in decreasing the Pn rate. Kobza and Edwards [34] as well as Law and Crafts-Brandner [17,34] suggested that Rubisco deactivation might be more important than altered thylakoid function in decreasing CO_2 _assimilation under high temperatures. Abdullaev et al. [35] has also reported that the reduction of CO2 photosynthetic assimilation could induce deactivation of Rubisco and other Calvin enzyme in high mountainous plant treated with UV-radiation.

Our results indicated that the ascorbate peroxidase activity significantly increases during different stages of the leaf life especially during the post-anthesis and post-maturation stages (Figure 9). It appears that the increase of ascorbate peroxidase activity acts as a defense mechanism against aging in plants. However, due to the oxidative stress induced by paraquat, independent of different doses, the enzyme activity decreased significantly (Figure 9). It has previously been shown that paraquat could decrease ascorbate peroxidase activity by 83% in pea plant [32]. It is speculated that the decrease of ascorbate peroxidase activity in wheat plant could be due to the sudden accumulation of H2O2 and the low level production of ascorbate peroxidase as a result of paraquat treatment (Figure 9).

## Conclusion

In spite of the reduction of the protein contents by proteases, Rubisco remains fully functional and retains its photosynthetic rate in high level. However, alteration in Rubisco conformation, specially the LUS subunit can decrease its functionality independently, which in turn reduces the photosynthetic rate significantly. The increment of Ci is in parallel with the reduction of G that is mainly due to the impairment of Calvin cycle and CO2 assimilation. This suggests a strong correlation between Ci and photosynthetic rate. Our results indicated that both aging and paraquat treatment as well as many other oxidative stresses could indirectly influence the degradation of Rubisco by impairing Mehler reaction or electron transfer chain which in turn reduces the activity of Rubisco by altering its conformation.

## Competing interests

The authors declare that they have no competing interests.

## Authors' contributions

GSH, MG and MM have made substantial contributions to conception and design, acquisition of data, and carried out the study and drafted the manuscript.

DN, MA, AA, and EC associated equally in completion of the project and performing the experiment.

All Authors read and approved the final manuscript
